# Transition from laparoscopic to robotic rectal resection: outcomes and learning curve of the initial 100 cases

**DOI:** 10.1007/s00464-020-07731-0

**Published:** 2020-06-18

**Authors:** Pim B. Olthof, Louis J. X. Giesen, Teddy S. Vijfvinkel, Daphne Roos, Jan Willem T. Dekker

**Affiliations:** 1 Department of Surgery, Erasmus Medical Center, Doctor Molewaterplein 40, 3015 GD Rotterdam, The Netherlands; 2 Department of Surgery, Reinier de Graaf Gasthuis, Delft, the Netherlands

**Keywords:** Rectal cancer, Rectal resection, Robotic, Robot-assisted surgery

## Abstract

**Background:**

Following several landmark trials, laparoscopic rectal resection has reached standard clinical practice. Current literature is undecided on the advantages of robotic rectal resection and little is known on its learning curve. This study aimed to compare the outcomes of the first 100 robotic rectal resections to the laparoscopic approach in a teaching hospital experienced in laparoscopic colorectal surgery.

**Methods:**

A retrospective analysis was conducted of a prospective cohort of all consecutive rectal resections between January 2012 and September 2019 at a single center. All laparoscopic cases were compared to the robotic approach. Outcomes included operative time, morbidity, anastomotic leakage, and hospital stay.

**Results:**

Out of the 326 consecutive resections, 100 were performed robotically and 220 laparoscopically, the remaining 6 open cases were excluded. Median operative time was lower for robotic cases (147 (121–167) versus 162 (120–218) minutes *P* = 0.024). Overall morbidity was lower in robotic cases (25% versus 50%, *P* < 0.001), while major morbidity was similar. Anastomotic leakage was observed in 11% (8/70) of robotic and 15% (18/120) of laparoscopic anastomoses, despite more anastomoses in the robotic group (70%, 70/100 versus 55%, 120/220, *P* = 0.001). Median length of stay was 4 (4–7) days after a robotic and 6 (5–9) days after a laparoscopic procedure.

**Discussion:**

Implementation of a robotic rectal resection program in an experienced laparoscopic surgery center was associated with reduced operative time, length of stay, and fewer complications despite a learning curve.

Laparoscopic resection has become the standard of care for patients with resectable rectal cancer. Several randomized trials have demonstrated faster recovery from laparoscopic surgery compared to an open approach with similar oncological outcomes [1–4]. These results have induced a surge in the proportion of patients treated laparoscopically over the last decade and the majority of rectal resections in the Netherlands are now performed minimally invasive [5, 6].

The development of minimally invasive surgery using robotic assistance allows surgeons to work in a comfortable position using articulating instrument arms with 3-dimensional vision. These features facilitate operating in the confined space of the pelvis. The robot-controlled camera is also stable which enhances sharpness of the image. All these characteristics are hypothesized to allow more accurate surgery. The limited randomized trial data comparing robotic and laparoscopic rectal resection show similar outcomes yet fewer conversions with the robotic approach, demonstrating robotic rectal surgery is at least as safe and non-inferior [7–10]. Long-term outcomes and functional patient reported outcomes are still lacking. However, the higher costs of robot-assisted surgery might question its relevance in the absence of a clear benefit for patients [11].

Learning a new technique such as robotic rectal resection takes time, and the learning curve is likely to effect the surgical outcomes. The aim of this study was to analyze the outcomes and the learning curve of a starting robotic rectal resection program in an experienced laparoscopic colorectal cancer center.

## Materials and methods

### Study design

All consecutive rectal resections for rectal cancer at the ‘Reinier de Graaf Gasthuis’ between January 1, 2012 and September 30, 2019 were included. Clinical data were obtained from the prospective Dutch ColoRectal Audit. Additional variables were retrospectively collected from the electronic medical records. The need for ethical approval and individual informed consent was waived by the institutional medical ethics committee.

### Patient work up and treatment

All patients were discussed in a multidisciplinary meeting to discuss the treatment regimen and treated according to the Dutch guidelines. All patients underwent a preoperative MRI of the Pelvis. Short course radiotherapy (5 × 5 Gy) was considered in case of cT1-3 cN1 stages and T3N0 with over 5 mm of extramural invasion. Chemoradiotherapy was considered in case of cT4, cT3 within 1 mm of the mesorectal margin, or cN2. In case of chemoradiotherapy, a MRI was performed after 6 weeks for response evaluation, followed by discussion in the multidisciplinary meeting.

All rectal cancer resections were performed with adherence to the total mesorectal resection principles. Up to 2016, all patients were operated laparoscopically unless this was considered contra-indicated. Anastomoses were created using circular stapling. Specimen extraction was performed using a small Pfannenstiel incision.

Staring from October 2016, robotic resections were implemented in a stepwise approach. After completing the Intuitive training program, all robotic resection were performed by two surgeons together. After being proctored for the first cases, robotic resection became the standard. A Da Vinci Surgical Systems Xi with a single console was used for all cases.

### Variables

Anastomotic leakage was defined as a defect of the intestinal wall at the anastomosis leading to communication between intra- and extra luminal compartments that required an intervention [12, 13]. Hospital stay was defined the number of days between surgery and discharge. Reoperation, re-intervention, and readmission for any reason were recorded within the first 30 days postoperatively. Procedure time was defined as the time from incision to wound closure. Operative time was defined as the total time a patient spends in the operating room. Mean visual analogue pain scores were recorded from 3 standard times of vital sign assessments during the first three days. Time to first stool was defined as the time from wound closure to recording of passing of the first stool. Estimated blood loss was calculated including gauze weight. When blood loss was too low to estimate it was set at 5 mL. A negative resection margin was defined as negative tumor-free resection margins at the distal, proximal, and circumferential margins and separate tumor deposits and lymph nodes were not considered part of the margin status [14].

All complications within 30 days after surgery were scored and graded according to the Dindo classification system and the comprehensive complications index (CCI) was calculated [15, 16]. Death within 90 days after surgery was defined as postoperative mortality.

### Statistical analysis

Categorical variables were displayed as numbers with percentages and differences were tested using Chi-square or Fishers exact tests. Continuous variables were displayed as medians with inter-quartile-range (IQR) with the exception of CCI, which was presented as mean with standard deviation. Differences were tested using Mann Whitney *U* tests. The predictive value of c-reactive protein level on postoperative day 3 for anastomotic leakage was analyzed using area under the curve (AUC) analysis. Using 2 × 2 tables the positive and negative predictive values were calculated. Learning curves were analyzed using the CUSUM method, in which the incidence of a particular event at the time of each case is plotted against the consecutive cases minus the expected incidence of the particular event. In the CUSUM analyses the median operative time and incidence of anastomotic leakage in the total laparoscopy group was used as expected incidence for the robotic cases. All statistical analyses were performed using SPSS (version 24.0, IBM, Chicago, IL).

## Results

During the study period 326 patients underwent a resection for rectal cancer. Six procedures (2%) were performed open and were excluded from the analyses. All 100 (31%) robotic procedures were performed after November 2016 and the remaining 220 (67%) procedures were laparoscopic, of which 15 (7%) were performed after initiation of the robotic program (Table S1). Two surgeons performed 92% of all 326 procedures, specifically 5 out of 6 open cases, 194 out of 220 laparoscopic cases, and together performed all robotic cases.

Patient characteristics of the cohort are displayed in Table 1. The cohorts differed in body mass index which was higher in the robotic group. There were slightly more distal tumors (≤ 3 cm at endoscopy) in the laparoscopic group (38; 17%) compared to the robotic group (9; 9%, *P* = 0.047). Using the robotic approach more low anterior resections were performed, so a greater proportion of anastomoses were created and diverting ileostomies were rare in both groups.

**Table 1:** Baseline characteristics of patients who underwent robotic or laparoscopic rectal resection

	Robotic (*n* = 100)	Laparoscopic (*n* = 220)	*P* value
Age, median (IQR)	68 (58–74)	69 (61–76)	0.148
Male sex, *n* (%)	63 (63)	141 (64)	0.900
Body mass index, kg/m^2^, median (IQR)	26.3 (24.2–29.2)	25.2 (23.2–28.4)	0.042
ASA score, *n* (%)			0.284
I	27 (27)	56 (26)	
II	60 (60)	120 (55)	
III	11 (11)	42 (19)	
IV	2 (2)	2 (1)	
Tumor height, *n* (%)			0.047
≤ 3 cm	9 (9)	38 (17)	
> 3 cm and ≤ 7 cm	13 (13)	40 (18)	
> 7 cm	78 (78)	142 (65)	
cT stage, *n* (%)			0.539
T1	1 (1)	6 (3)	
T2	26 (26)	63 (29)	
T3	70 (70)	148 (67)	
T4	3 (3)	3 (1)	
cN stage, *n* (%)			0.767
N0	47 (47)	94 (43)	
N1	28 (28)	65 (30)	
N2	25 (25)	61 (28)	
cM stage, *n* (%)			0.761
M0	97 (97)	210 (96)	
M1	3 (3)	10 (5)	
Mesorectal margin < 1 mm, *n* (%)	18 (18)	41 (19)	1.000
Neoadjuvant treatment, *n* (%)			0.216
Short course radiotherapy (5 × 5 Gy)	26 (26)	75 (34)	
Chemoradiotherapy	28 (28)	68 (31)	
Procedure, *n* (%)			0.001
Low anterior resection	70 (70)	120 (55)	
Hartmann	11 (11)	65 (30)	
Abdominoperineal resection	19 (19)	35 (16)	
Diverting ileostomy, *n* (%)	2 (2)	12 (6)	< 0.001

The postoperative outcomes are summarized in Table 2. The total required operative time was lower in the robotic group (*P* = 0.031) and the robotic procedures were performed a median 15 min faster compared to the laparoscopic approach (*P* = 0.024). The estimated blood loss associated with robotic procedures was too low to estimate in most cases. The only conversion with robotic resection was in the beginning of the experience compared to 19 (9%) with laparoscopy. The robotic conversion was performed due to a lack in progression due to confined space in the pelvis in the presence of abundant intra-abdominal adipose tissue. While overall complications were less frequent with the robotic approach (25% versus 50%, *P* < 0.001), major complications and anastomotic leakage were similar. Postoperative recovery was faster after robotic resection, demonstrated by shorter length of stay, fewer readmissions, and higher rate of textbook outcomes.

**Table 2:** Postoperative outcomes of patients who underwent robotic or laparoscopic rectal resection

	Robotic (*n* = 100)	Laparoscopic (*n* = 220)	*P* value
Overall operative time, min, median (IQR)	203 (172–230)	214 (173–277)	0.031
Procedure duration, min, median (IQR)	147 (121–167)	162 (120–218)	0.024
Blood loss, mL, median (IQR)	5 (5–5)	50 (5–150)	< 0.001
Conversion, *n* (%)	1 (1)	19 (9)	0.006
Negative resection margins, *n* (%)	99 (99)	213 (97)	0.443
Resected lymph nodes, median (IQR)	13 (11–17)	12 (10–16)	0.006
Any complication, *n* (%)	25 (25)	110 (50)	< 0.001
Major complication, *n* (%)	18 (18)	46 (21)	0.651
CCI, median (IQR)	0 (0–6)	4 (0–30)	0.001
Anastomotic leakage, *n* (%)	8 (11)	18 (15)	0.662
Reoperation, *n* (%)	17 (17)	32 (15)	0.616
Readmission, *n* (%)	9 (9)	36 (16)	0.053
Length of stay, days, median (IQR)	4 (4–7)	6 (5–9)	< 0.001
Text book outcome, *n* (%)	72 (72)	107 (49)	< 0.001
90-day mortality, *n* (%)	0 (0)	1 (1)	1.000

In the first 62 robotic cases, a robotic stapler (45 mm) was used and 44 anastomoses (71%) were created. The anastomotic leakage rate in these patients was 16% (7/44). After these cases the robotic staplers were discontinued in favor of laparoscopic staplers (60 mm). In the subsequent 48 robotic cases, 26 anastomoses were created (54%) with an anastomotic leakage rate of 4% (1/26). This difference did not reach statistical significance (*P* = 0.243).

The time to passage of first flatus and stool was recorded for all patients. The median time to flatus was 34 (21–54) h after a robotic and 28 (16–51) h after a laparoscopic procedure (*P* = 0.087). Median time to stool in all patients, including colostomies, was 63 (35–88) h after robotic and 53 (27–82) h after laparoscopic surgery (*P* = 0.076). For patients with an anastomosis, the median time to flatus was shorter in the laparoscopic group (34 (23–54) versus 25 (16–44) h, *P* = 0.011), while time to first stool was similar (63 (35–83) versus 56 (28–78) hours, *P* = 0.113).

The number of harvested lymph nodes was a median 13 (11–17) in the robotic group and 12 (10–16) in the laparoscopy group (*P* = 0.006). A positive resection margin were found in 1 (1%) robotic case and 7 (3%) laparoscopic cases. The positive margin in the robotic case was a positive circumferential margins, while 3 out of the 7 were circumferential in the laparoscopic cases and the remaining 4 at a positive at the distal margin.

**Fig. 1:**
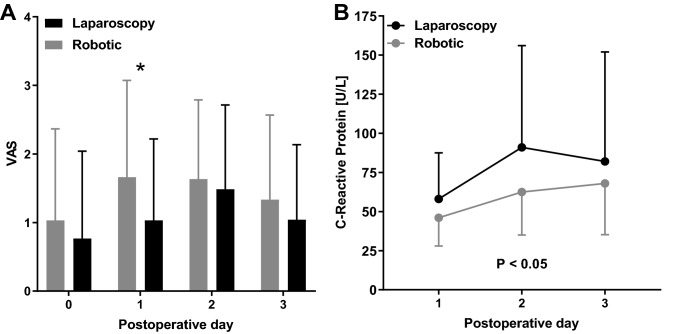
**A** Mean visual analogue pain scores on the first three postoperative days. * indicated *P* < 0.05 between groups. **B** Mean postoperative CRP levels

The mean pain scores were slightly higher at postoperative day one in robotic cases, however, the mean difference was small with a mean (SD) of 1.7 (1.4) compared to 1.0 (1.2) which is likely not clinically significant (Fig. 1). Mean C-reactive protein levels were lower after robotic resections. This resulted in increased accuracy to predict anastomotic leakage with the postoperative day 3 c-reactive protein level in the patients who were operated robotically compared to the laparoscopic approach. The AUC value for day 3 c-reactive protein level was 0.90 (0.80–1.00) in the robotic cases resulting in a positive predictive value of 50% when CRP is 124 U/L or higher and a negative predictive value of 98%. In the laparoscopic cases the AUC was 0.82 (0.72–0.92). In these patients the 124 U/L cut-off would have resulted in a positive predictive value of 28% and a negative predictive value of 91%.

**Fig. 2:**
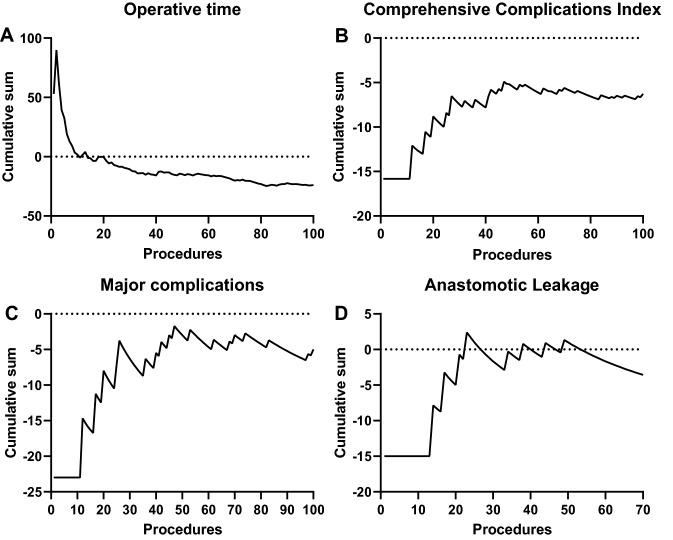
CUSUM learning curves of the robotic cases for **A** operative time, **B** the comprehensive complications index, **C** and major complications in all patients, and for **D** anastomotic leakage in only patients with an anastomosis. The incidence of the respective variable in all laparoscopic cases in the present cohort was used as reference for the robotic learning curve

When looking at the learning curve of the first 100 robotic cases using the CUSUM method, operative time greatly decreased over the first 40 cases after which the decrease continues but more gradually (Fig. 2). From the 20th procedure the mean robotic operative time was lower than that of the laparoscopic cases. A similar stabilization after 40 procedures could be observed for the CCI which stabilized but remained below the mean CCI observed with laparoscopy. The major complication rate appeared to stabilize after the first 40 cases. The curve was calculated for anastomotic leakage for patients with an anastomosis and also stabilized after 30 to 40 cases.

## Discussion

In a single center series of 326 patients, 320 (98%) underwent a minimally invasive resection of which 92% were operated by two single surgeons. In the 100 patients who underwent a robotic procedure, operative time, blood loss, conversion rate, overall complication rate, length of stay, and readmission rate were all in favor of the robotic approach compared to the laparoscopic procedures. Major complications and anastomotic leakage were similar. These results demonstrate that a robotic rectal resection program can be implemented in an experience laparoscopic clinic with favorable outcomes despite a clear learning curve.

The ROLARR trial is the largest randomized trial comparing robotic with laparoscopic rectal resection [7]. Conversion to laparotomy was the primary endpoint which was not different between the laparoscopic and robotic study arms after randomization of 471 patients. None of the secondary endpoints showed a difference. The 1% conversion rate in the current series is lower compared to the 8% ROLARR robotic conversion rate [9]. A potential explanation could be the learning curve. In the ROLARR trial surgeons were required to perform at least 10 robotic resections for study participation, while the learning curve analyses in Fig. 2 show operative time, which is often used to estimate the learning curve [17], stabilizes around 30 cases. A more recent meta-analysis showed a conversion rate more similar to the current series of only 2% after robotic resection compared to 7% with laparoscopy in almost 5000 patients [10].

The anastomotic leakage rate is reported to be 2–12% after robotic rectal resection and usually around 11% after laparoscopic rectal resection [18, 19]. All comparative studies failed to show a difference in anastomotic leakage rate between the laparoscopic and robotic approach [9, 20, 21]. These rates are most likely dependent on numerous factors including the tumor height, patient selection, neoadjuvant treatment, use of diverting ileostomy, and the definition used for anastomotic leakage. In the present study, the use of ileostomy was low in the robotic group with 2%, while the proportion of patients with an anastomosis increased from 65% in the laparoscopic to 86% in the robotic group when excluding abdominoperineal resections. Although the anastomotic leakage rate was 11% in the robotic group and comparable to the 15% in the laparoscopic procedures, the robotic approach has led to creating anastomosis in a greater proportion of patients. Considering more patients received an anastomosis, including the more high risk cases, the leakage rate might be relatively low, but such an effect is hard to objectify.

During the initiation of the robotic program it was decided to discontinue the robotic stapler due to a subjective high number of required stapler fillings, in conjunction with a relatively high leakage rate all at the posterior side. The trusted laparoscopic stapler was used through a trocar at the future Pfannenstiel specimen extraction side as opposed to the right robotic port. This new approach led to a lower rate of anastomotic leakage. While this did not reach statistical significance, if the 4% leakage rate after robotic resections using laparoscopic staples continues, this will likely result in a statistically significant difference compared with laparoscopy. Although it cannot be excluded that these results can partially be attributed to a learning curve.

This study has some limitations, mostly secondary to the retrospective study design which is subject to bias, yet the data were extracted form a prospective database. All consecutive resections were included and after the first case of robotic resection, only 15 patients were operated laparoscopically and 3 open resulting in 83% robotic resections. The laparoscopic cases after initiation of the robotic program were all due to logistical reasons (e.g., mostly related to availability of the robotic system). Furthermore 92% of patients were operated by two surgeons that used the same laparoscopic techniques and performed all robotic cases together, which likely results in a more homogenous cohort compared to other studies.

In conclusion, the implementation of a robotic rectal resection program in an experienced laparoscopic center resulted in shorter operative times, fewer conversions, more primary anastomoses, fewer complications, and shorter hospital stay. Although major complications including anastomotic leakage were similar, the comparison of outcomes are in the presence of a clear learning curve of around 40 cases in this study and are therefore likely to further improve over time. Future studies should include functional and long term outcomes, as well as cost-effectiveness to establish the definitive place of robot-assisted rectal resections in clinical practice.
